# How Much Should a City Pay Its Health Officer?

**Published:** 1889-11

**Authors:** 


How Much should a City Pay Its Health Officer?—The Michigan State Board of Health has recently published a paper by its Secretary, Dr. H. B. Baker, in which he asks the question: How much can the average city or village afford to pay its Health Officer? He answers this question in this way:

Statistics, which cannot be questioned, prove that in those localities in Michigan where the recommendations of the State Board of Health are carried out, about eighty per cent. of the deaths from diphtheria and scarlet fever are prevented by the thorough isolation of all infected persons, and the thorough disinfection of all infected persons, things, and places. Statisticians usually value a person in the prime of life as worth to the community about $1,000.

Dr. Baker thinks that, in a village of 1,500 inhabitants, a health officer can easily save the lives of two children and one grown person in each year, and he concludes that such a village can well afford to pay its health officer $2,000 for the prevention and restriction of scarlet fever, diphtheria, and typhoid fever—and make money by the transaction.

---

At the recent election in France, twenty-two physicians were elected on the first ballot for seats in the Chamber of Deputies. The second final ballot will elect as many more, it is predicted.

